# Supplementary material for: Specific Norovirus Interaction with Lewis x and Lewis a on Human Intestinal Inflammatory Mucosa during Refractory Inflammatory Bowel Disease
Source: mSphere. 2021 Jan 13;6(1):e01185-20. doi: 10.1128/mSphere.01185-20 (PMC7845605; doi:10.1128/mSphere.01185-20)
Supplement: TABLE S1 [file mSphere.01185-20-st001.pdf]

Table S1

Crohn's Disease								
No	Anatomical site	HES stain	VLP	ABO	Le^a	sLe^a	Le^x	sLe^x
1	Ileum	QM (80%)	100%-PM	100%-PM	100%-PM	30%-GC	80%-GC	10%-BC
		RM (20%)	100%-PM	100%-PM	100%-PM	100%-PM	90%-PM	90%-PM
2	Ileum	QM (100%)	100%-PM	100%-PM	20%-GC	20%-GC	50%-GC	10%-BC
3	Ileum	QM (80%)	100%-PM	100%-PM	30%-GC	30%-GC	40%-GC	10%-BC
		RM (20%)	100%-PM	100%-PM	80%-PM	80%-PM	100%-PM	100%-PM
4	Ileum	QM (100%)	100%-PM	100%-PM	30%-GC	30%-GC	30%-GC	10%-BC
5	Ileum	QM (100%)	100%-PM	100%-PM	30%-GC	30%-GC	20%-GC	5%-BC
6	Ileum	QM (30%)	100%-PM	100%-PM	30%-GC	30%-GC	40%-GC	10%-BC
		RM (70%)	100%-PM	100%-PM	80%	80%	90%-PM	90%-PM
7	Ileum	QM (90%)	100%-PM	100%-PM	10%-GC	10%-GC	30%-GC	5%-BC
		RM (10%)	100%-PM	100%-PM	90%	90%	80%-PM	80%-PM
8	Proximal Colon	QM (100%)	100%-PM	100%-PM	100%-GC	5%-GC	70%-GC	5%-BC
9	Proximal Colon	QM (100%)	100%-PM	100%-PM	60%-GC	5%-GC	70%-GC	5%-BC
10	Proximal Colon	QM (100%)	100%-PM	100%-PM	100%-GC	1%-GC	50%-GC	1%-BC
11	Transverse Colon	QM (80%)	30%-GC	0%	30%-GC	30%-GC	30%-BC	5%-BC
		RM (20%)	90%-PM	0,5%-E	90%-PM	90%-PM	80%-PM	80%-PM
12	Transverse Colon	QM (100%)	50%-BC	0%	80%-GC	20%-GC	70%-GC	5%-BC
13	Transverse Colon	QM (50%)	20%-BC	0%	70%-GC	5%-GC	10%-GC	5%-BC
		RM (50%)	100%-PM	0,5%-E	90%-PM	90%-PM	100%-PM	100%-PM
14	Transverse Colon	QM (100%)	20%-BC	0%	100%-PM	5%-GC	30%-BC	5%-BC
15	Sigmoid	QM (100%)	20%-BC	0%	50%-GC	5%-GC	30%-BC	5%-BC
16	Sigmoid	QM (90%)	20%-BC	0%	10%-GC	10%-GC	20%-BC	5%-BC
		RM (10%)	100%-PM	0,5%-E	100%-PM	100%-PM	100%-PM	100%-PM
