# Supplementary material for: Specific Norovirus Interaction with Lewis x and Lewis a on Human Intestinal Inflammatory Mucosa during Refractory Inflammatory Bowel Disease
Source: mSphere. 2021 Jan 13;6(1):e01185-20. doi: 10.1128/mSphere.01185-20 (PMC7845605; doi:10.1128/mSphere.01185-20)
Supplement: TABLE S2 [file mSphere.01185-20-st002.pdf]

Table S2

Ulcerative Colitis								
Patient	Anatomical site	HES stain	VLP	ABO	Le[a]	sLe[a]	Le[x]	sLe[x]
17	Sigmoid	QM (100%)	30%-BC	0%	80%-GC	70%-GC	30%-BC	20%-BC
18	Sigmoid	QM (90%)	60%-GC	0%	50%-GC	50%-GC	30%-BC	5%-BC
		RM (10%)	90%-PM	0,5%-E	80%-PM	80%-PM	100%-PM	100%-PM
19	Sigmoid	QM (100%)	20%-BC	0%	90%-GC	30%-GC	10%-BC	10%-BC
20	Sigmoid	RM (100%)	90%-PM	0%	90%-PM	90%-PM	80%-PM	80%-PM
21	Sigmoid	QM (30%)	20%-BC	0%	50%-GC	50%-GC	30%-GC	10%-GC
		RM (70%)	100%-PM	0%	70%-PM	70%-PM	80%-PM	80%-PM
22	Sigmoid	RM (100%)	100%-PM	0%	100%-PM	100%-PM	90%-PM	90%-PM
23	Rectum	QM (100%)	5%-BC	0%	0%	0%	40%-BC	5%-BC
24	Rectum	RM (100%)	90%-PM	0%	100%-PM	100%-PM	90%-PM	90%-PM
25	Rectum	QM (60%)	10%-BC	0%	5%-GC	5%-GC	20%-BC	10%-BC
		RM (40%)	90%-PM	0,5%-E	50%-PM	50%-PM	90%-PM	90%-PM
