# Supplementary figures and images for: Specific Norovirus Interaction with Lewis x and Lewis a on Human Intestinal Inflammatory Mucosa during Refractory Inflammatory Bowel Disease
Source: mSphere. 2021 Jan 13;6(1):e01185-20. doi: 10.1128/mSphere.01185-20 (PMC7845605; doi:10.1128/mSphere.01185-20)

**Figure S1**

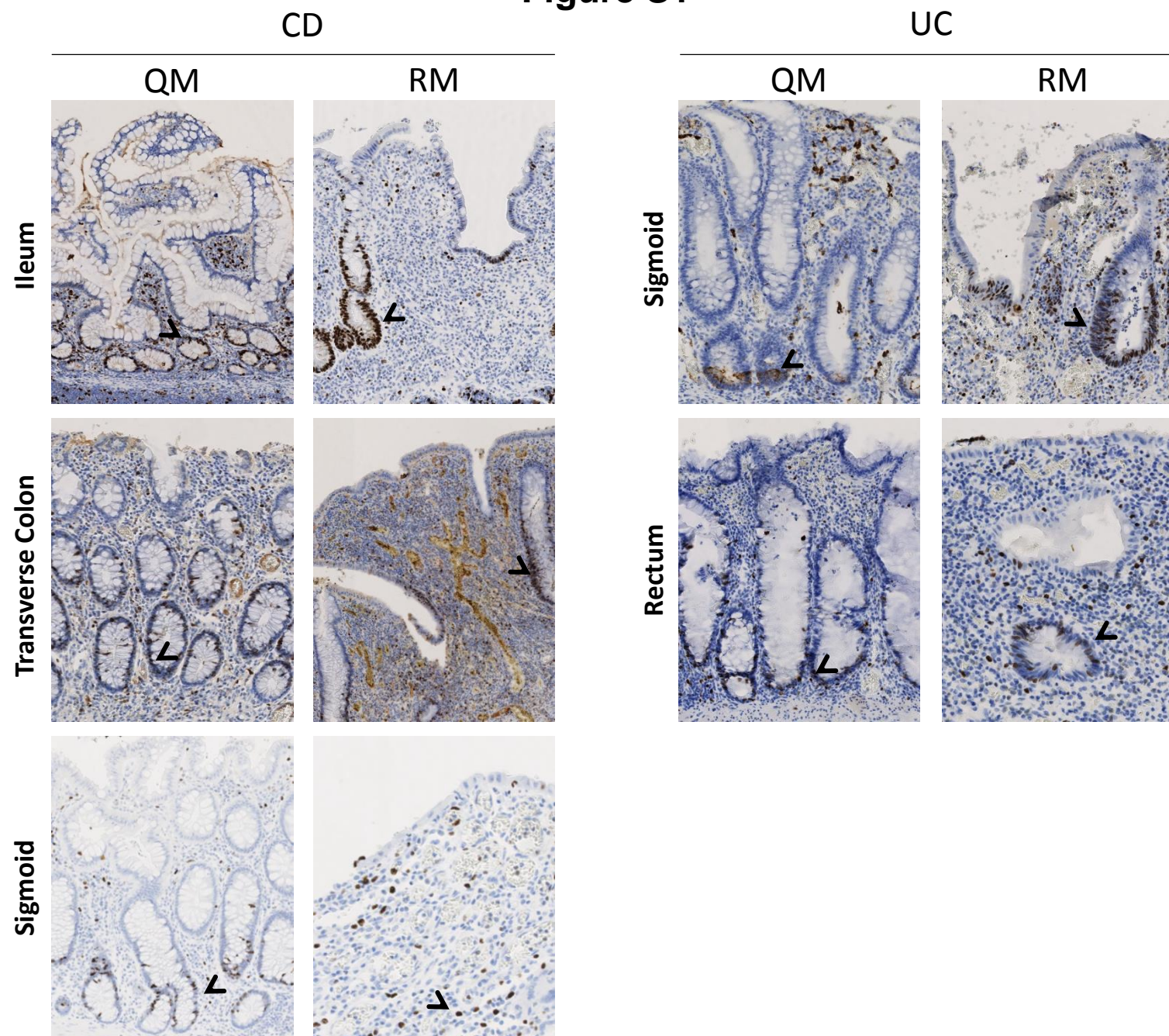

Supplement: FIG S1 [file mSphere.01185-20-sf001.pdf]

**Figure S2**

Mock

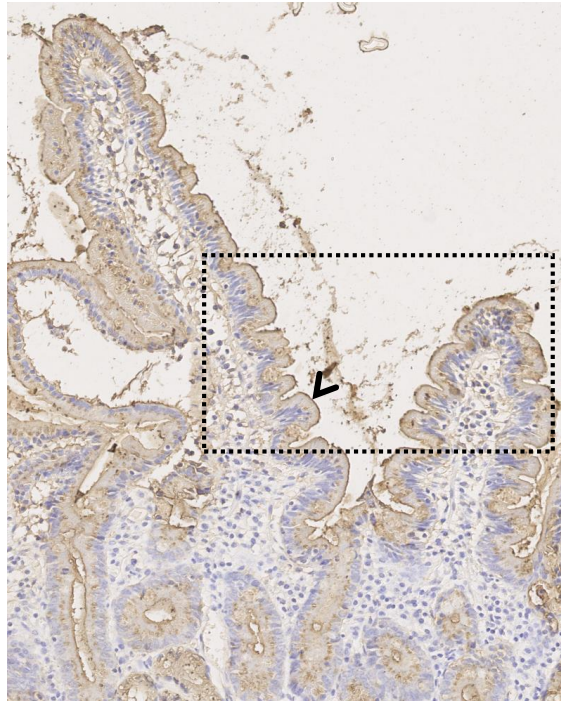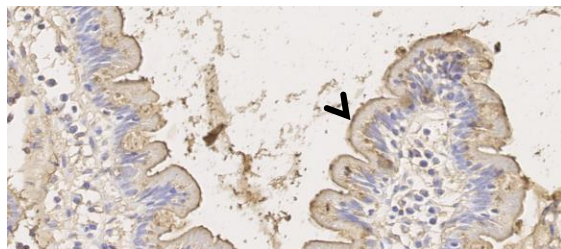

HPA

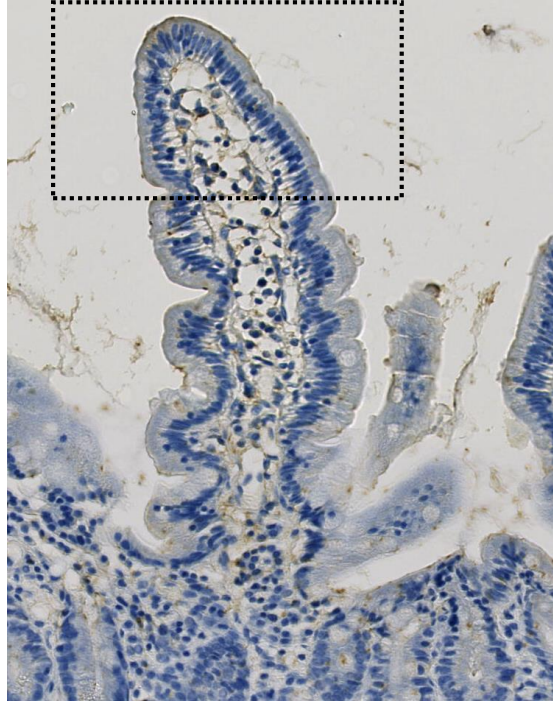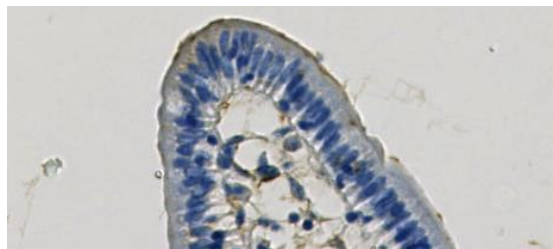

Boiled HPA

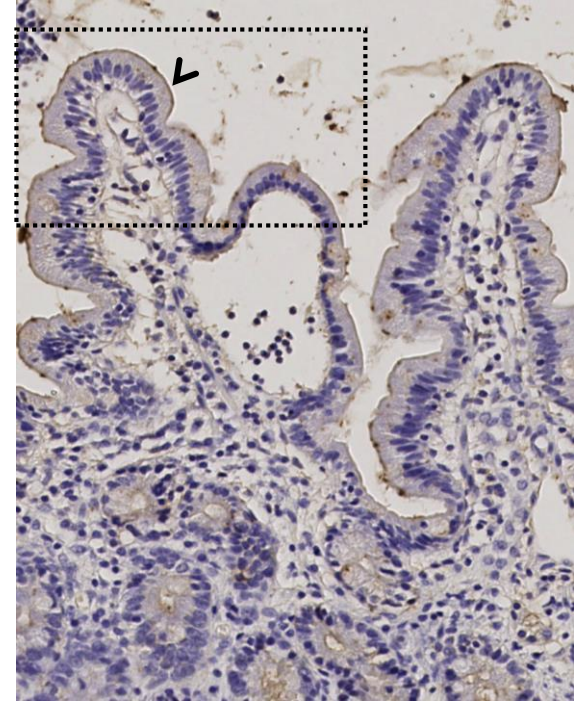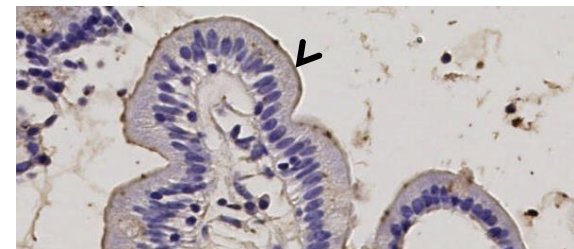

Supplement: FIG S2 [file mSphere.01185-20-sf002.pdf]

Figure S3

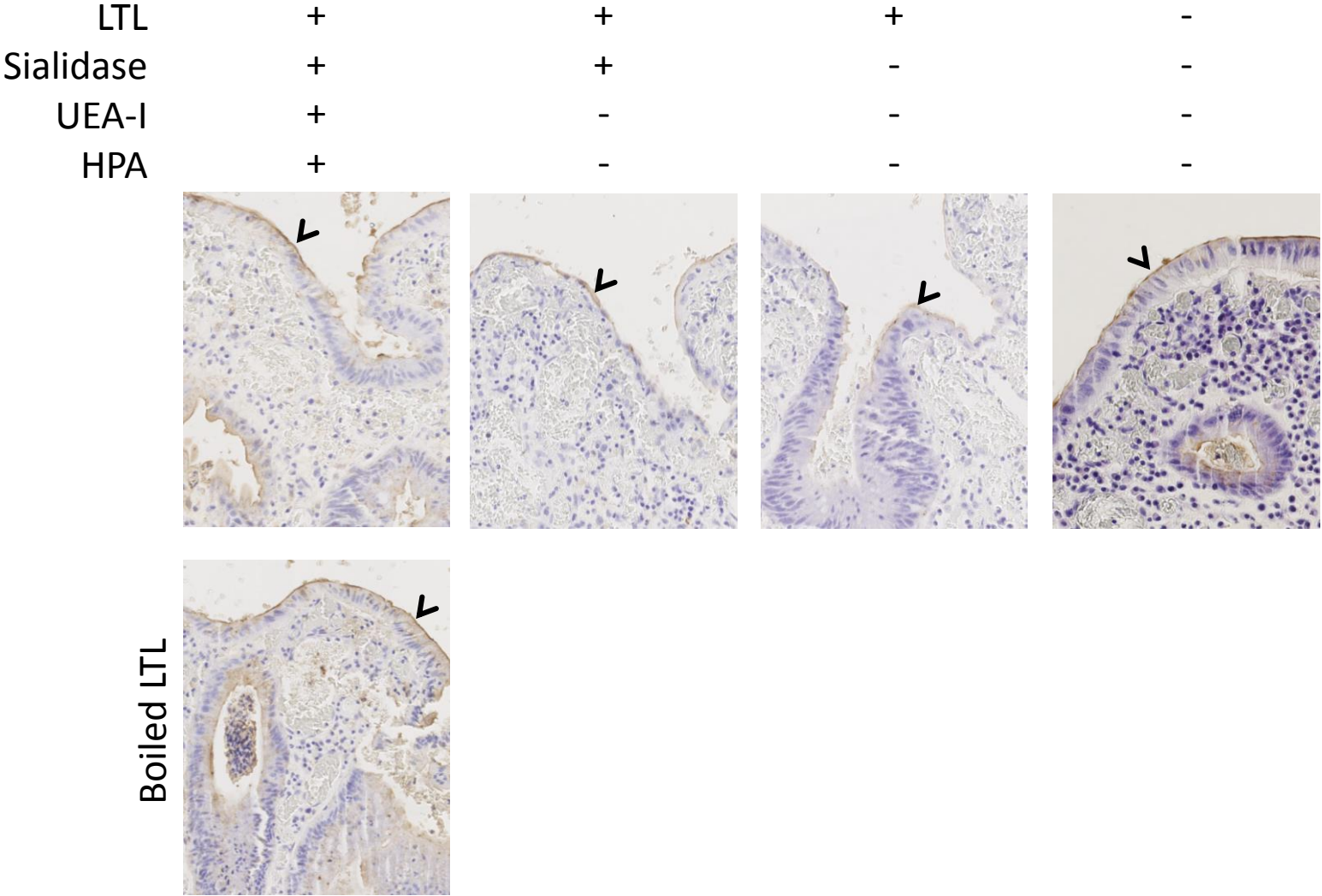

Supplement: FIG S3 [file mSphere.01185-20-sf003.pdf]

**Figure S4**

CD (*Patient 13*)

UC (*Patient 22*)

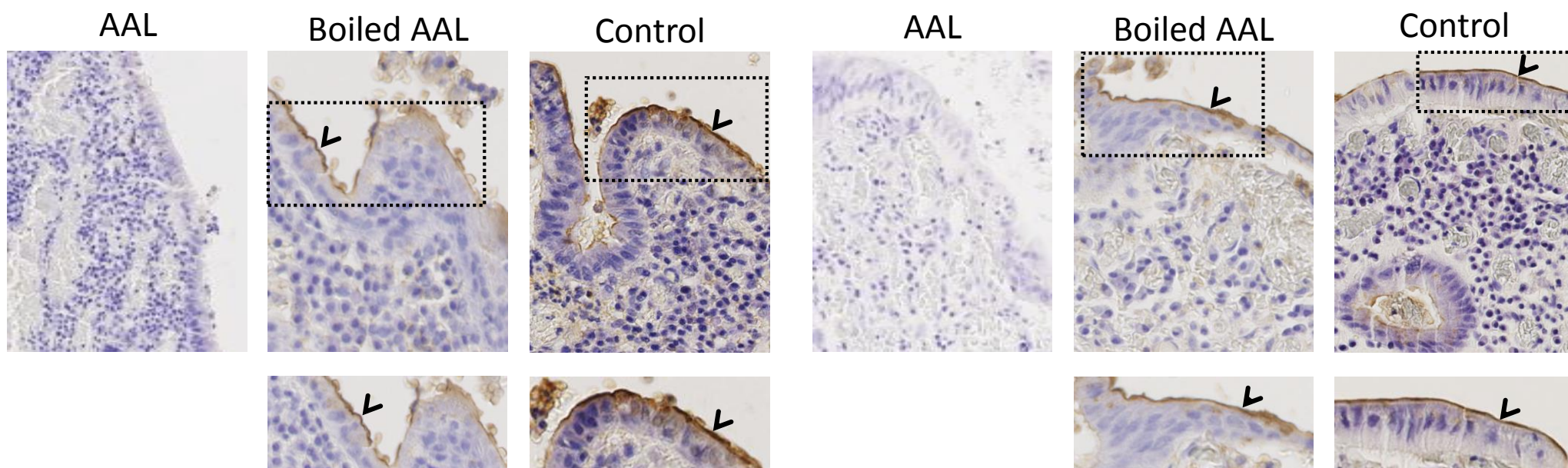

Supplement: FIG S4 [file mSphere.01185-20-sf004.pdf]

**Figure S5**

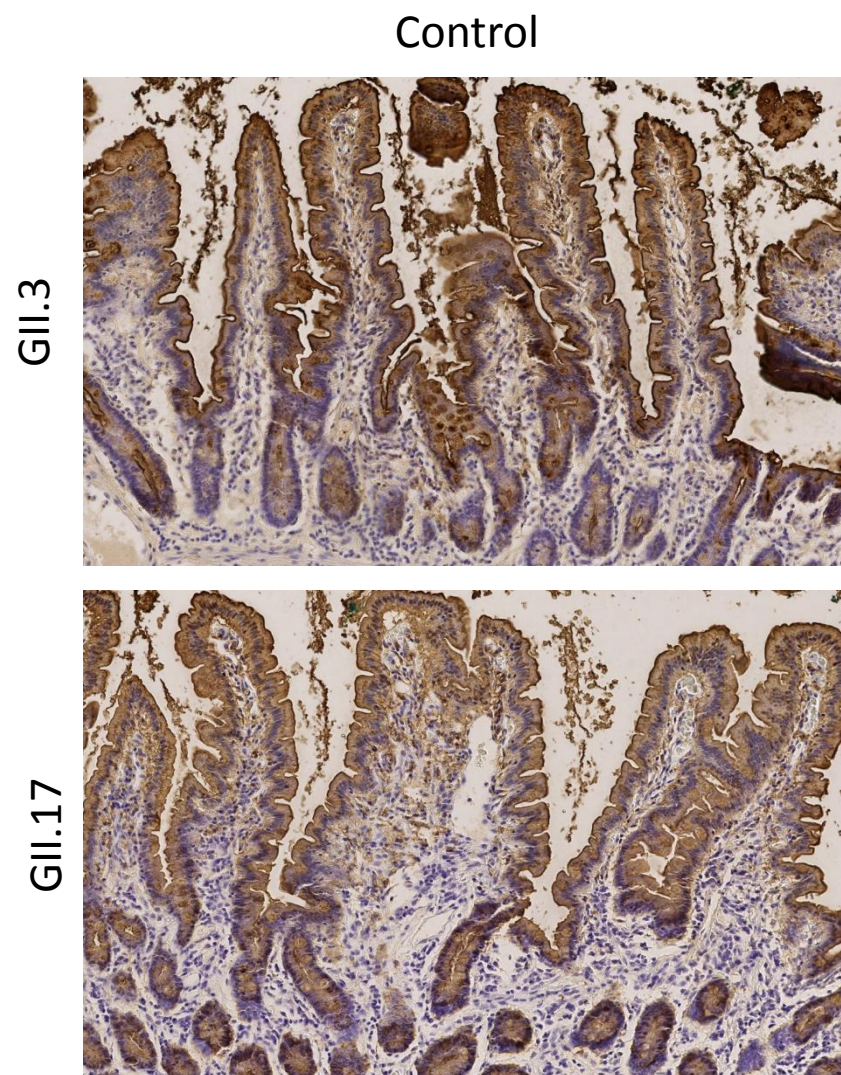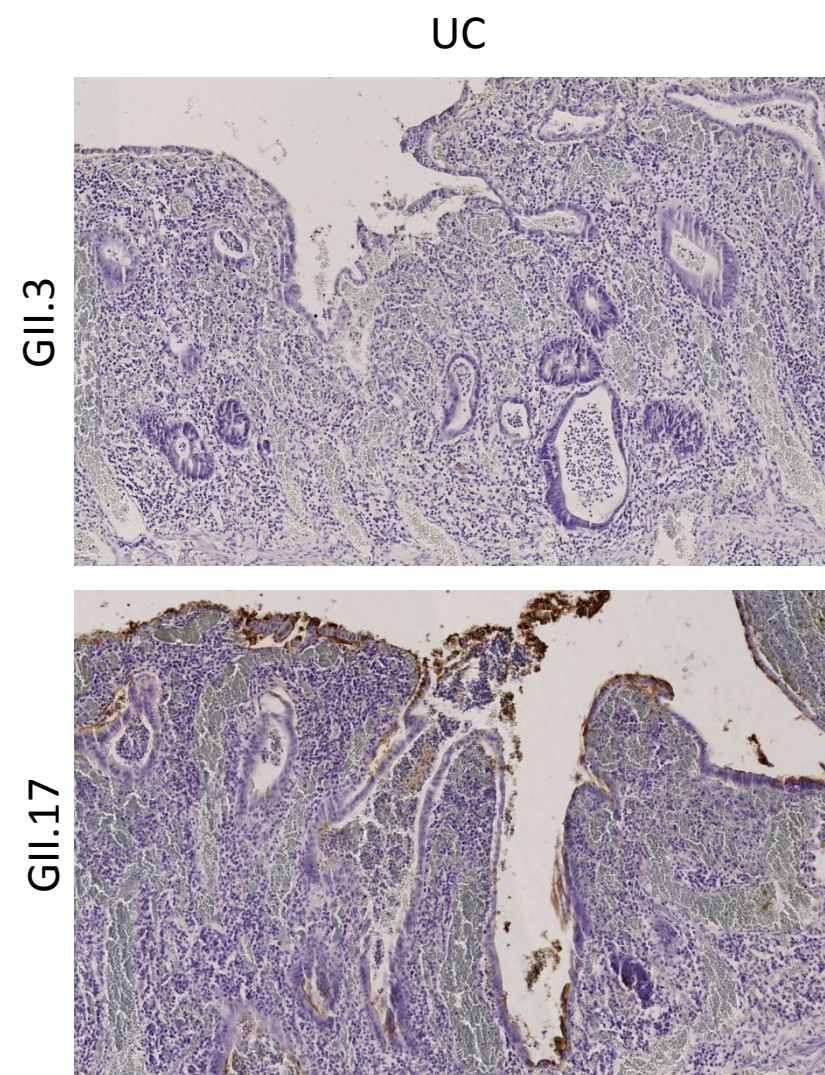

Supplement: FIG S5 [file mSphere.01185-20-sf005.pdf]

**Figure S6**

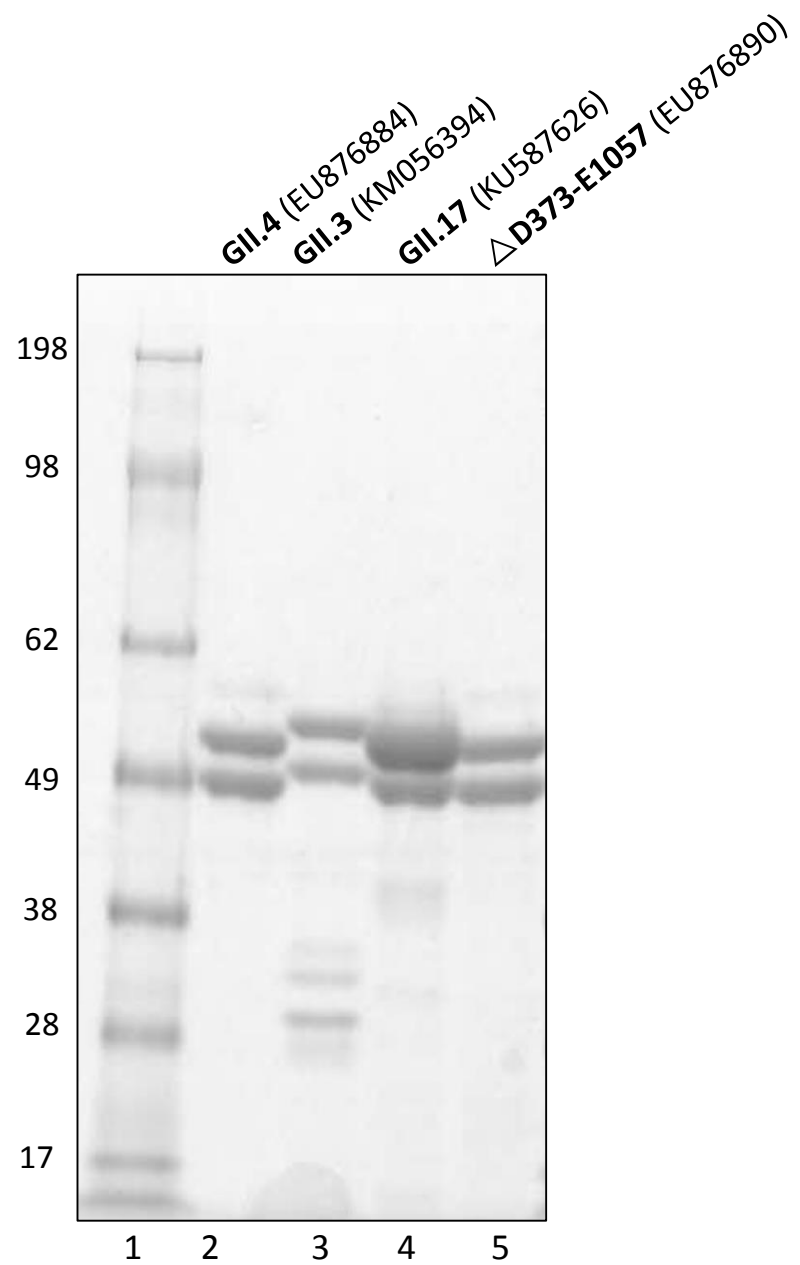

Supplement: FIG S6 [file mSphere.01185-20-sf006.pdf]
